# Multiorgan sequelae following non-COVID-19 respiratory infections: a review

**DOI:** 10.1007/s15010-025-02519-7

**Published:** 2025-04-04

**Authors:** Barbara Christine Weckler, Max Kutzinski, Claus Franz Vogelmeier, Bernd Schmeck

**Affiliations:** 1 Department of Medicine, Pulmonary and Critical Care Medicine, Clinic for Airway Infections, University Medical Centre Marburg, Philipps-University Marburg, Marburg, Germany; 2 Institute for Lung Research, Universities of Giessen and Marburg Lung Center (UGMLC), Philipps-Universität Marburg, Marburg, Germany; 3 Member of the CALM-QE network, Marburg, Germany; 4 Department of Medicine, Pulmonary and Critical Care Medicine, University Medical Centre Marburg, Philipps-University Marburg, Marburg, Germany; 5 German Centre for Lung Research (DZL), Marburg, Germany; 6 Core Facility Flow Cytometry– Bacterial Vesicles, Philipps-University Marburg, Marburg, Germany; 7 Institute for Lung Health (ILH), Giessen, Germany; 8 German Centre of Infectious Disease Research, Marburg, Germany; 9 Centre for Synthetic Microbiology (Synmikro), Philipps-University Marburg, Marburg, Germany

**Keywords:** Respiratory infections, Post-acute infection sequelae, Comorbidities, Long-term sequelae

## Abstract

**Background:**

While numerous studies have documented severe and long-term health impacts of COVID-19 infections on various organs, the prolonged multisystemic implications of other acute respiratory infections (ARIs) are poorly understood. This review therefore analyzed currently available studies about these sequelae of ARIs excluding COVID-19.

**Main body:**

Multiple pathogens causing ARIs are associated with significant long-lasting impairments across various organ systems. Cardiovascular events occur in 10–35% of patients following ARIs, with an elevated risk persisting for 10 years. The stroke incidence ratio increases significantly after ARIs up to 12.3. Pulmonary sequelae are common, including abnormal lung function in 54%, parenchymal opacification in 51%, lung fibrosis in 33–62%, asthma in 30%, and bronchiectasis in 24% of patients. The risk of developing dementia is increased 2.2-fold. Posttraumatic stress disorder, depression, anxiety, and chronic fatigue occur in 15–43%, 15–36%, 14–62%, and 27–75% of patients, respectively. 28-day mortality from CAP with (versus no) additional cardiovascular event is increased to 36% (versus 10%). Long-term mortality from CAP (versus no CAP) remains elevated for years post-infection, with a 1-year, 5-year, and 7-year mortality rate of 17% (versus 4%), 43% (versus 19%), and 53% (versus 24%), respectively. Patients´ quality of life is significantly reduced, with 17% receiving invalidity pensions and 22% retiring within 4 years of severe ARIs.

**Conclusion:**

Non-COVID-19 ARIs are associated with clinically relevant, frequent, and long-term sequelae involving multiple organ systems. Further prospective studies are needed.

## Background

Acute respiratory tract infections (ARIs) are major causes of morbidity and mortality worldwide [1]. Defined as infectious diseases of the respiratory tract, ARIs can be caused by a variety of viral or bacterial pathogens [2]. ARIs can be classified into upper and lower respiratory tract infections, with the anatomical boundary between these categories being the vocal cords [3]. Common upper respiratory tract infections include rhinitis, sinusitis, pharyngitis, epiglottitis, laryngitis, and otitis media, while bronchitis and pneumonia are lower respiratory tract infections. The severity of ARIs can range from asymptomatic or mild, self-limiting conditions to severe or fatal disease [2]. The risk of sequelae following ARIs seems higher in patients requiring hospitalizations compared to mild ARIs [4]. For more than one hundred years, several outbreaks of viral respiratory infections have occurred, including the Influenza pandemics of 1889 and 1892 (Russian Flu), the Spanish Flu Pandemic in 1918–1919, the poliomyelitis epidemic in Los Angeles in 1934 [5], severe acute respiratory syndrome (SARS) in 2003 [6], Influenza A subtype H1N1 in 2009 [7], Middle East respiratory syndrome (MERS) in 2012 [8], and COVID-19 in 2019 [9]. Respiratory infections may also be caused by bacteria, of which Streptococcus pneumoniae is most common in patients with CAP [10]. The growth in the world population and urbanization [11], the high extent of global travelling [12], the rise in migration [12], and the climate change [13] contribute to the risk of future outbreaks of infectious diseases including respiratory infections. Following ARIs, a significant proportion of patients experience persistent or new symptoms. These lingering health issues have been defined as “post-acute infection sequelae” [14]. These sequelae can lead to chronic disability, impacting patients’ long-term health and quality of life [15–46].

As a consequence of the COVID-19 pandemic, many studies have analyzed complications following ARIs with severe acute respiratory syndrome coronavirus 2 (SARS-CoV-2). In contrast, evidence about the complications following ARIs other than COVID-19 is scarce. This is owing to the lack of uniform diagnostic criteria for post-acute infection sequelae as this prevents clinicians from systematically including patients affected in studies. Whether diseases that have been diagnosed after respiratory tract infections are linked to post-acute infection sequelae or correspond to manifestations of previously latent, preexisting diseases is unclear. Even though the need for further studies analyzing cardiovascular and pulmonary complications following respiratory infections has been identified as such [47, 48], the role of respiratory infections other than COVID-19 for the development of secondary diseases is poorly understood as yet. Previous studies have analyzed the effects of ARIs on individual organ systems only without considering the broader systemic implications. Based on an extensive literature research, this article provides current knowledge of post-acute infection sequelae involving multiple organ systems following non-COVID-19 ARIs. This review focuses on the etiology, type, epidemiology, and timing of complications following respiratory infections including their impact on mortality, quality of life, and burden on society.

## Main text

### Etiology and assumed pathomechanisms of post-acute infection sequelae

A wide range of ARIs is associated with subsequent chronic disability in patients. Table 1. summarizes the non-SARS-CoV-2 pathogens of ARIs for which post-acute infection sequelae have been documented in the literature.

**Table 1:** Pathogens causing acute respiratory infections (other than COVID-19) associated with post-acute infection sequelae

Type of pathogen	Name of pathogen of acute respiratory infections with evidence of post-acute infection sequelae
**Viruses**	Cytomegalovirus [15, 16]
	Coxsackievirus B [17, 18]
	Hantavirus [19, 20]
	Human immunodeficiency virus [21, 22]
	influenza [23, 24]
	Measles virus [25, 26]
	Middle East respiratory syndrome coronavirus [27, 28]
	Mycobacterium tuberculosis [29, 30, 31]
	Rhinovirus [32, 33]
	Respiratory syncytial virus [33, 34]
	Severe acute respiratory syndrome coronavirus [35, 36]
	Varicella zoster virus [37, 38]
**Bacteria**	Chlamydia pneumoniae [39, 40]
	Coxiella burnetii [41, 42]
	Legionella pneumophila [43, 44]
	Streptococcus pneumoniae [45, 46]

The development of chronic sequelae after respiratory infections involves complex interactions between the infectious agent, the host´s immunological defenses, and the impacted organ systems. These interactions can lead to organ-specific structural defects, such as pulmonary fibrosis, systemic consequences including cardiovascular complications, and unexplained post-acute infection sequelae [49]. These diverse outcomes underscore the far-reaching and often unpredictable ramifications of respiratory infections, extending well beyond the acute phase of illness.

Several pathomechanisms have been postulated [49]. These include a continuous stimulation of the immune system by pathogen remnants, such as viral RNA or bacterial cell wall [50] with ongoing engagement of B and T lymphocytes and subsequent persistent inflammation [49], autoimmune activation [51] as a direct result of the immune system targeting the pathogen or as a bystander effect unrelated to pathogen structure, and microbiota-gut-brain axis dysregulation [49]. Post-acute infection sequelae may be related to an inability to repair tissue damage caused by the infection and subsequent immunopathologic effects. For example, vascular damage and lung fibrosis that is not repaired properly in the course of the acute respiratory infection, can result in long-term respiratory dysfunction [52]. Furthermore, mitochondrial dysfunction has been proposed to contribute to the emergence of post-acute infection sequelae. Mitochondria are responsible for energy production in the cell required to maintain many critical cellular processes [53]. Viral infections can lead to mitochondrial dysfunction with subsequent impact on metabolism, inflammation, oxidative stress [54], cell proliferation [55], apoptosis [55, 56], antiviral responses [57], and aging [58, 59]. SARS-CoV-2 RNA can reside in the mitochondria of the host [60, 61]. Cardiac mitochondrial disruption after infection with SARS-CoV-2 contributes to cardiovascular dysfunction in COVID-19 patients [62] and is associated with neuropathology [63]. The underlying pathomechanisms of post-acute infection sequelae are not fully understood. The etiopathogenesis of post-acute infection sequelae is likely multifactorial, involving a complex interplay of various physiological processes. Future research will be essential to elucidate the complete spectrum of pathways involved and their relative contributions to the observed clinical manifestations.

### Post-acute infection sequelae and their multisystemic impact

**Fig. 1:**
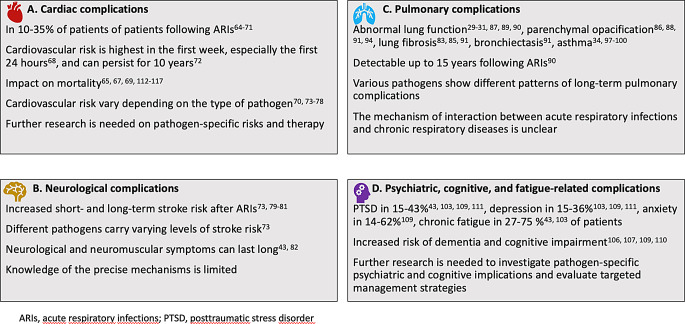
At a glance: Organ manifestations of post-acute infection sequelae other than long COVID*

ARIs can have wide-ranging effects on various organ systems. Figure 1 provides an overview of key insights into the specific organ manifestations of post-acute infection sequelae other than long COVID.

The following sections detail ARI-related complications by organ system.

### Cardiovascular complications following CAP

**Fig. 2:**
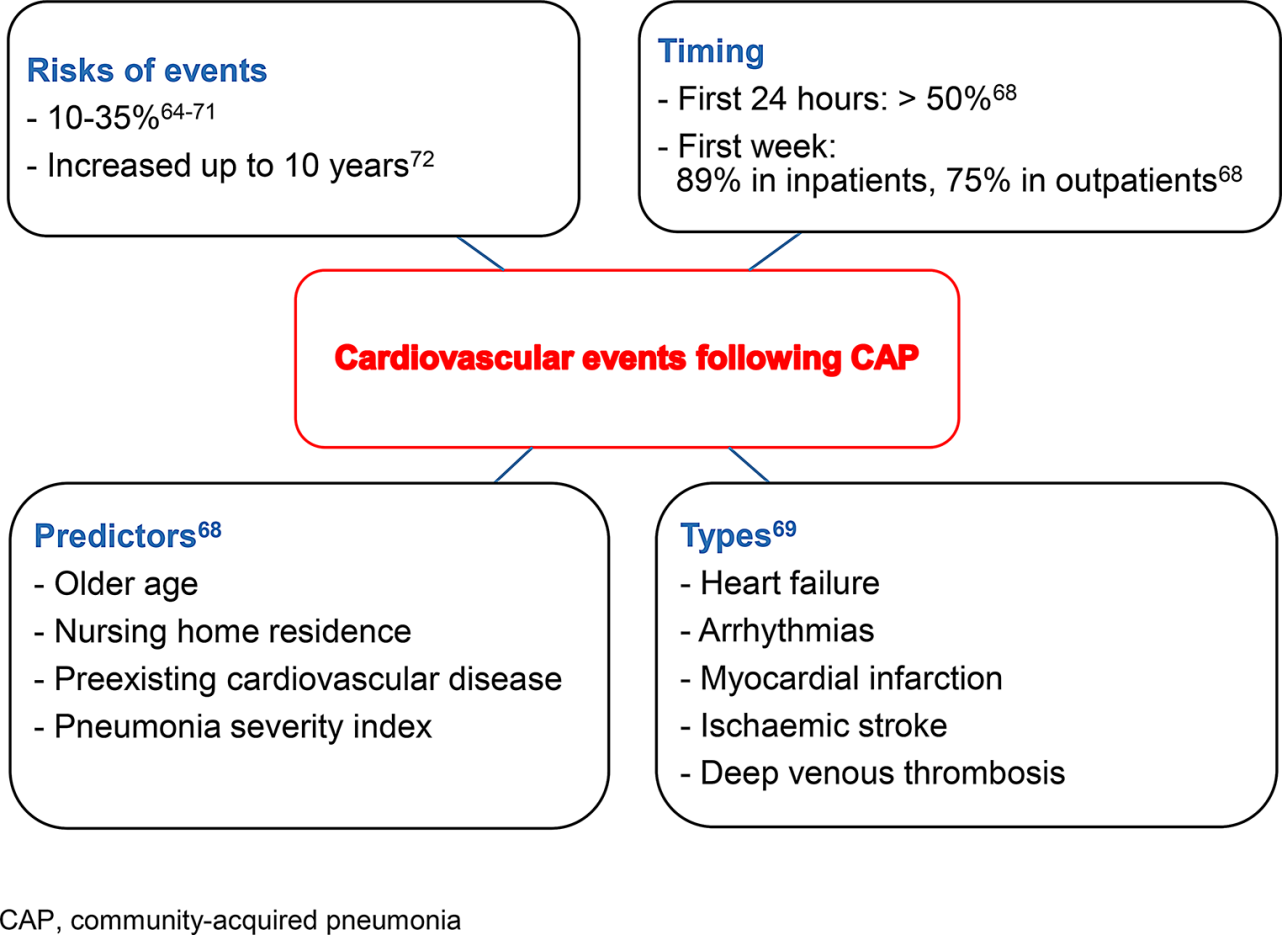
Cardiovascular events following community-acquired pneumonia

The risk of cardiovascular events following CAP varies widely, ranging from 10 to 35% [64–71]. Figure 2 provides crucial findings about these complications following ARIs. They comprise plaque-related (e.g., myocardial infarction) and plaque-unrelated cardiac events (e.g., heart failure, arrhythmias) [66]. A prospective analysis [68] of 1,343 inpatients and 944 outpatients with CAP shows incidental cardiac complications including new or worsening heart failure, arrhythmias, or myocardial infarction in 358 (26.7%) inpatients and 20 (2.1%) outpatients. Importantly, the vast majority of cardiac events (89.1% in inpatients, 75.0% in outpatients) is diagnosed within the first week following disease onset, with more than half of them identified in the first 24 h. Predictors of their occurrence are older age, nursing home residence, preexisting cardiovascular disease, and pneumonia severity. Intrahospital cardiovascular events including heart failure, atrial fibrillation, myocardial infarction, ischemic stroke, and deep venous thrombosis happen in 380 (32.2%) of 1,182 patients with CAP [69]. The risk of cardiovascular events remains elevated for 10 years following CAP [72].

CAP: community-acquired pneumonia.

Several studies have examined the pathogen-specific incidence of cardiac complications following respiratory infections:

#### Streptococcus pneumoniae

In hospitalized patients (*n* = 170), 19.4% experience ⩾1 major cardiac event (myocardial infarction, atrial fibrillation, ventricular tachycardia, new-onset or worsening congestive heart failure) at admission, with a 7.1% incidence of myocardial infarction of [70]. Warren-Gash et al. report a 5.98-fold increase in the age- and season-adjusted myocardial infarction incidence within 1–3 days of Streptococcus pneumoniae infection [73]. Another study comparing the incidence of cardiovascular events between hospitalized pneumococcal (*n* = 66) and non-pneumococcal pneumonia (*n* = 64) within one year after hospital admission reveals a higher rate of acute coronary syndrome in patients with pneumococcal pneumonia (19.7% versus 1.6%; *p* = 0.001), whereas the rates of arrhythmias and heart failure are not different [74].

#### Influenza virus

Among patients hospitalized with influenza infection, approximately one in eight (11.7%) experience severe heart complications [75]. The most prevalent of these are acute heart failure and acute ischemic heart disease in 6.2% and 5.7%, respectively. Admissions for acute myocardial infarction are increased six-fold (20.0 admissions per week versus 3.3 admissions per week) within the first week after influenza diagnosis [76]. The age- and season-adjusted incidence ratio is 9.8 for myocardial infarction on days 1–3 following influenza virus infection [73]. A meta-analysis [77] of case-control studies revealed a significant association between recent influenza infection, influenza-like illness, or respiratory tract infection and the occurrence of myocardial infarctions. The pooled odds ratio was 2.0, indicating that individuals with these infections were approximately twice as likely to experience myocardial infarctions. Importantly, the same study [77] demonstrated that influenza vaccination offered significant protection against acute myocardial infarctions. Vaccinated individuals experienced a 29% lower risk of myocardial infarctions compared to those who were unvaccinated. This finding underscores the potential of influenza vaccination as an important preventive measure against cardiovascular events.

#### Respiratory syncytial virus (RSV)

A recent comprehensive study [78] shows that 22.4% of adults aged 50 years or older with RSV infection experience cardiac events during hospitalization. The most prevalent complication is acute heart failure, affecting 15.8% of patients. Other cardiac events include acute ischemic heart disease in 7.5%, hypertensive crisis in 1.3%, ventricular tachycardia in1.1%, and cardiogenic shock in 0.6% of patients. Another study corroborates the increased risk of cardiac complications associated with RSV infection, demonstrating an incidence ratio of 3.5 for acute myocardial infarction within 7 days following RSV detection [76].

#### Other respiratory viruses

The number of admissions for acute myocardial infarction is elevated within the 7 days of diagnosis for adenovirus, coronavirus, enterovirus (including rhinovirus), parainfluenza virus, and human metapneumovirus [76].

### Stroke

Respiratory tract infections are a risk factor for cerebrovascular events [73, 79–81]. Clayton et al. [79] report that the risk of stroke increases by 92% in the first week after respiratory infections. Rates of stroke after respiratory infections with *Streptococcus pneumoniae* and respiratory viruses (including *influenza virus*,* parainfluenza virus*,* rhinovirus*,* RSV*,* human metapneumovirus*) are elevated with day 1–3 age- and season-adjusted incidence ratios of 12.3 and 6.79, respectively [73]. A population-based self-controlled case series study found that the risk of atherothrombotic events (ischemic stroke and acute myocardial infarction) increases 2-fold during the 14 days after mild influenza infection, and more than 4-fold after severe influenza infection in vulnerable patients [81]. Stroke rates following both bacterial and viral infections continue to be increased until 28 days following the initial infection [73]. Chen et al. [80] demonstrate that patients with pneumococcal pneumonia have a 3.65-fold higher risk of stroke during the first year compared to those without pneumonia, after adjusting for confounders. This corresponds to an additional 14% stroke risk (*p* = 0.032) in these patients. Although the elevated stroke risk decreases during the second year, it remains significantly higher than in individuals who had not experienced pneumococcal pneumonia [80].

### Neurological complications other than stroke

RSV, influenza virus, coronavirus, and the human metapneumovirus can lead to a wide range of neurological manifestations including seizures (both febrile and afebrile), status epilepticus, encephalopathies, and encephalitis [82]. The detection of these viruses in cerebrospinal fluid suggests that, following initial infection in the respiratory tract, these pathogens have the potential to disseminate systemically and ultimately infiltrate the central nervous system. A follow-up study [43] of 122 survivors of *Legionella pneumophila* infection shows neurological symptoms in 66% and neuromuscular symptoms in 63% of patients 17 months after disease onset.

### Pulmonary complications

**Fig. 3:**
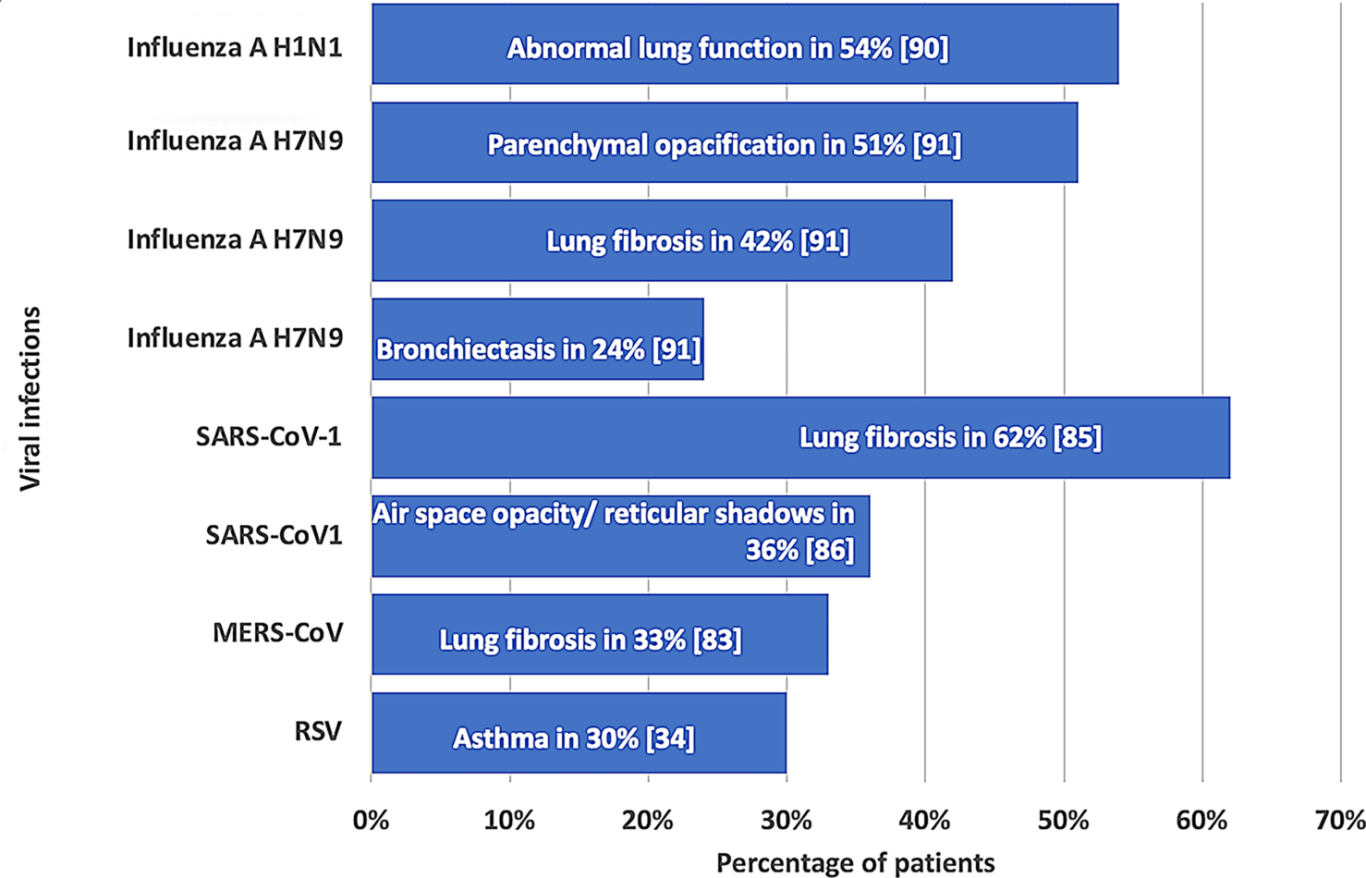
Long-term pulmonary sequelae of viral respiratory infections

Multiple studies have documented a correlation between ARIs and both immediate and lasting pulmonary consequences [34, 35, 43, 83–93]. Figure 3 gives key findings about these pulmonary complications post-ARIs.

The following section offers a comprehensive overview of the evidence related to various respiratory pathogens and their pulmonary effects.

#### Influenza virus

Long-term pulmonary sequelae following influenza A virus infections have been documented in several studies. A study on influenza A virus subtype H1N1 infection demonstrates that 54.2% (26/48) of patients exhibit abnormal pulmonary function approximately one year after recovery and hospital discharge [90]. In the case of influenza A virus subtype H7N9, computed tomography of the chest performed 12 months post-infection reveals signs of lung fibrosis and parenchymal opacification (ground-glass opacities and reticular patterns) in 41.5% and 51.2% of patients, respectively [91]. Further pathological findings on computed tomography of the chest include bronchiectasis in 24.4%, pneumatocele in 9.8%, small bullous cysts in 4.9%, nodules in 9.8%, and pleural thickening in 22.0% of patients [91].

#### MERS-CoV

Evidence shows that 12 (33%) of 36 patients exhibit lung fibrosis on chest X-ray [83] 32–230 days after recovery from the acute infection.

#### Mycobacterium tuberculosis

Several studies [94–96] investigating long-term pulmonary sequelae on radiograph after tuberculosis infection reveal a range of parenchymal sequelae associated with Mycobacterium tuberculosis infection, including fibrotic changes, calcifications, atelectasis, emphysema, and bronchiectasis and lymph node enlargement. Research about long-term pulmonary function impairments following tuberculosis infection found that 24.6% of patients exhibit reduced forced expiratory volume in 1 second six months after pulmonary tuberculosis infection [29]. Another recent study [30] demonstrates restrictive and obstructive patterns on spirometry taken at 19 months after tuberculosis diagnosis in 36.5% and 2% of patients, respectively. Corroborating these findings, a systematic review and meta-analysis of spirometry data from 14,621 people reveals that at least 10–15% of tuberculosis survivors experience severe lung impairment six months following diagnosis [31]. These results collectively underscore the potential for tuberculosis to cause lasting damage to respiratory function, emphasizing the need for long-term monitoring and management of patients following tuberculosis.

#### RSV and rhinovirus

RSV infections in infancy is associated with a higher asthma prevalence at the age of 7 ½ years (30% versus 3% in the control group; *p* < 0.001) [34]. Such an RSV infection related increase in the asthma prevalence was confirmed in several prospective long-term follow-up studies [97–100]. Rhinovirus is a risk factor for asthma [32, 97, 101, 102]. The mechanisms by which RSV, rhinovirus, and both personal and environmental factors promote the development of asthma have not been fully understood and warrant further research [93].

#### SARS-CoV-1

An early follow-up study of patients with SARS reveals that 62% of patients display signs of pulmonary fibrosis on thin-section computed tomography 37 days after hospital admission [85]. 35.8% and 30% of patients demonstrate pathological findings (abnormal scores in airspace opacity and /or reticular shadows) on computed tomography 3 and 6 months post-infection, respectively [86]. A research study on asymptomatic teenagers (median age of 14.7 years) shows that 41.2% of them have residual ground glass opacification and/or air trapping on computed tomography at 6 months after serological diagnosis of SARS-Co-1 [88]. 1 year after hospitalization due to infection with SARS-CoV-1, lung diffusion capacity is impaired in 23.7% of patients [87], and the six-minute walking distance is reduced in 18% of patients [35]. Absolute and mass related peak oxygen consumption is significantly lower despite a normal lung function in asymptomatic teenagers (versus healthy controls) at 15 months after the serological confirmation of SARS-CoV-1 infection [88]. Two years after infection with SARS-CoV-1, 18.2%, 16.4%, 10.9%, and 52.7% of patients have reduced (< 80% of predicted values) FEV1, FVC, TLC, and DLCO, respectively [89]. Although the mean six-minute walking distance increases significantly (*p* = 0.016) between 3 and 6 months after disease onset, it continues to stay below reference data up to 2 years [89]. Three years following infection with SARS-CoV-1 [90], more than 1 of 5 (21.74%) patients displays a restrictive ventilation pattern, and more than one third (34.78%) of patients has a mildly reduced diffusion capacity (70–80% of the predicted value). 15 years after infection with SARS-CoV-1, the diffusion capacity is reduced in 38.46% of patients [90].

### Psychiatric, cognitive, and fatigue-related complications

Multiple studies (43, 103, 104, 105, 106, 107, 108, 109) have demonstrated a notable correlation between ARIS and psychiatric long-term sequelae. Figure 1D provides an overview of important insights into these complications. This section presents essential findings, structured by specific pulmonary diseases and different pathogens of ARIs.

#### Pneumonia

Multiple studies have proven an increased risk of cognitive decline and dementia following hospitalization for pneumonia. One analysis reveals that people previously hospitalized for pneumonia have a 53% higher incidence of cognitive impairment and dementia compared to matched controls [110]. The impact of pneumonia appears to be age-dependent because it was stronger in individuals between 45 and 60 years [110]. Another study [106] reports that pneumonia requiring hospitalization is associated with a 2.2-fold increased risk of developing dementia. This was confirmed by another study showing that patients hospitalized for pneumonia develop subsequent cognitive impairment and, in addition, depressive symptoms [107]. Also, this study did not find significant differences in cognitive impairment or depressive symptoms between survivors of pneumonia and those who had experienced myocardial infarction or stroke. These findings underscore the significant long-term impact of severe respiratory infections on cognitive function.

#### Acute lung injury

Twelve months following acute lung injury, survivors experience significant cognitive and psychiatric morbidities. Memory is impaired in 13% of patients, verbal fluency in 16%, and executive function in 49% of survivors at 12 months [109]. Depression, posttraumatic stress disorder (PTSD), and anxiety occur in 36%, 39%, and 62% of survivors, each [109]. In patients with severe CAP and acute respiratory distress syndrome (ARDS), delirium is common, affecting 88% of patients during their stay on intensive care unit [108]. The duration of delirium correlates with impaired cognitive performance in motor skills, memory function, and learning efficiency, as assessed 50 ± 6 months post-discharge from intensive care unit [108]. Furthermore, 16% of patients develop PTSD, with a positive correlation between the length of the delirium and the incidence of PTDS (median delirium duration 7 days in patients with PTDS versus 2 days in patients without PTDS; *p* < 0.027) [108].

#### SARS-CoV-1

Within 3.5 years post-infection, 42.5% of patients experience active psychiatric illness including PTSD in 54.5%, depression in 39.0%, somatoform pain disorder in 36.4%, panic disorder in 32.5%, chronic fatigue syndrome in 27%, and obsessive compulsive disorder in 15.6% of cases [103]. A meta-analysis [104] including twenty original studies about the psychological impact of SARS in survivors confirms high rates of PTSD, anxiety, and depression for years post-infection.

#### MERS-CoV

A substantial proportion of survivors exhibits psychiatric symptoms for an extended period following their initial infection. 12 months after infection with MERS, 42.9% and 27.0% of survivors reported PTSD and depression, respectively [111]. Further research revealed persistent mental health issues at both 12 and 18 months: At 12 months, 48.1% of patients experience chronic fatigue, 26.9% depression, and 42.3% PTSD, respectively [103]. By 18 months, these figures decreased to 32.7% for chronic fatigue, 17.31% for depression, and 26.9% for PTSD [105].

#### Legionella pneumophila

17 months after disease onset, PTSD and fatigue were present in 15% and 75% of patients, each [43].

### Mortality related to postinfectious sequelae

Numerous studies [65, 67, 69, 112–117] have shown a link between complications following ARIs and increased mortality rates.

28 -day mortality from hospitalized CAP with (versus no) additional cardiovascular event is 36% (versus 10%) (*p* < 0.001) [67]. 30-day mortality from CAP and subsequent cardiovascular event is fivefold (17.6% versus 4.5%; *p* < 0.001) [69] and twofold higher (16.3% versus 8.9%; *p* = 0.0001) [65] in comparison to patients hospitalized for CAP without cardiovascular event. 90-day mortality from CAP with cardiovascular and cerebrovascular event is increased, with an odds ratio of 1.93 for acute myocardial infarction, 2.39 for atrial fibrillation, and 1.79 for stroke [114]. Cardiac ischemia is the third leading cause of 90-day mortality after neurological disorders and malignancy in patients with CAP [115].

**Fig. 4:**
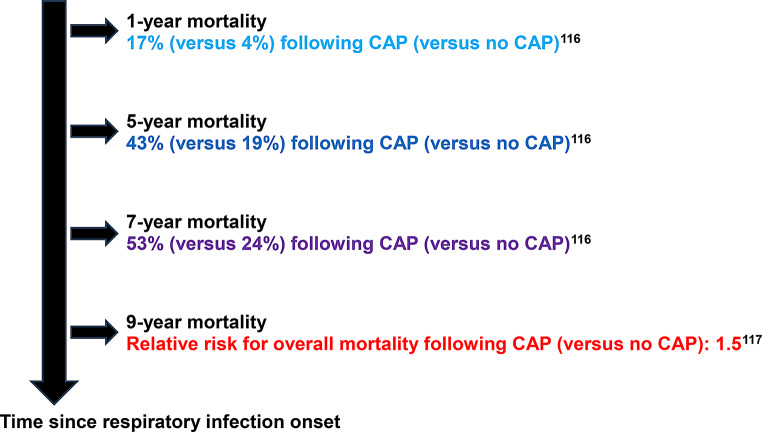
Long-term mortality following acute respiratory infections

After initial recovery from CAP (versus no CAP), cumulative 1-year, 5-year, and 7-year mortality rates are 17% (versus 4%), 43% (versus 19%), and 53% (versus 24%), respectively [116]. A 9.2-year follow-up study showed increased relative risks for overall mortality (RR: 1.5), pneumonia-related mortality (RR: 2.1), and cardiovascular mortality (RR: 1.4) [117]. Figure 4 offers an overview of long-term mortality based on time elapsed since respiratory infection onset.

#### Pathogen-specific mortality

Among patients who survive pneumococcal pneumonia for at least one month, nearly one-third (32.2%) die within the following decade [118]. The mean potential life loss is 9.9 years over a 30-year follow-up period in patients after invasive pneumococcal disease [119]. 30-day mortality following admission for infection with infuenza A, influenza B, RSV, rhinovirus, and human metapneumovirus accounts for 9%, 11%, 10%, 8%, and 9%, respectively [120], without significant differences in mortality between these pathogens. 30-day mortality in patients with influenza infection (versus age- and sex-matched individuals without influenza infection) is 4-fold higher [121], with an excess mortality rate ranging from 10.6 to 49.5 per 100,000 people [121–123].

These studies demonstrate the medium- and long-term mortality effects of ARIS. Notably, cardiovascular events in the context of CAP have been shown to increase mortality further. However, the specific contributions of the initial respiratory infection itself versus associated complications to this increased mortality are not clearly distinguishable. The specific role of the different pathogens causing respiratory infections is also still to be determined. Further research is crucial to address these gaps in knowledge, with a focus on identifying pathogen-specific complications and establishing tailored management strategies.

### Quality of life

Patients often experience a decline in their overall well-being after ARIs [35, 43, 86, 108, 124]. Research shows that individuals with CAP (versus non-diseased people) have a significantly lower quality of life [124] for 1 year post-infection as quantified by the Medical Outcomes Study Short Form 36-item health survey [SF-36, 125, 126], the EuroQol-5 Dimension [ Eq. 5D, 127], and Quality Adjusted Life Years [128].

Survivors of SARS experience a significant reduction in SF-36 scores at both 3 [35] and 6 months [86] after disease onset, indicating a sustained decline in health-related quality of life. Even though some improvement is noted in physical role, social function, and emotional role domains one year after hospital discharge, these areas do not return to normal levels [35]. One year after hospital discharge, general health, vitality, and social functioning domains remain 1.0, 0.8, and 0.8 standard deviations below the reference range, respectively, and quality of life continues to be reduced [35] according to the St George’s Respiratory Questionnaire [129].

The physical dimension of the health-related quality of life (HRQoL) as per SF-12 is significantly lower in critically ill patients with ARDS in the context of severe community-acquired pneumonia than in age- and sex-matched healthy controls (*p* < 0.0001) at 50 ± 6 months after discharge from intensive care unit [108]. Interestingly, while non-H1N1 associated severe CAP with ARDS (versus healthy controls) is associated with a reduction (*p* < 0.0001) in the physical dimension of the HRQoL as measured with the Medical Outcomes Study 12-Item Short Form [SF-12, 130], H1N1-related severe CAP with ARDS does not affect this dimension [108].

Legionella pneumophila infection survivors report significantly lower (*p* < 0.01) scores for 7 of the 8 dimensions of the SF-36 than an age- and sex-matched reference population [43] 17 months post-infection.

These data demonstrate that quality of life often declines after respiratory infections and that this effect can persist for months or years. Various infections may influence quality of life differently. As current evidence is limited, more research into pathogen-specific long-term consequences of ARIs on patients´ quality of life is needed.

### Burden on society

ARIs impose a significant burden on society [35, 89, 108] due to their frequent and long-lasting ramifications. Following CAP with ARDS, 17% of patients receive an invalidity pension, and 22% are retired 50 ± 6 months after discharge from intensive care unit [108]. 17% of patients with SARS do not resume work, and additional 9% of patients do not return to their pre-SARS level of work at 1year post-infection [35]. Another study demonstrates that 22% of patients do not return to work 2 years after infection with SARS-CoV-1 [89]. Recent evidence [131] suggests that seasonal influenza causes a greater burden of health loss in the post-acute phase compared to the acute phase of infection.

These findings substantiate considerable cumulative long-term implications of respiratory infections on society due to health loss with subsequent reduced ability to work. This highlights the need for improving patient management strategies to minimize or prevent adverse outcomes following respiratory infections.

### Common traits and specifics of different post-acute infection sequelae-related organ complications

Two common threads emerge across these complications: they occur frequently and often persist for extended periods. This pattern of widespread and long-lasting effects on multiple organs underscores the significant impact of ARIs on patients´ long-term trajectories. Nevertheless, the knowledge of the precise timeline, underlying mechanisms, and pathogen-specific contributions to multi-organ complications following ARIs remains limited.

### Implications of current evidence for everyday patient care

Because of the high rate of cardiovascular complications in the early phase of the infection, patients with ARIs may benefit from initial telemonitoring. Given the high incidence of postinfectious sequelae which can develop over years following ARIs, regular patient follow-ups appear crucial to identify complications in time. Interdisciplinary collaboration seems mandatory for providing comprehensive and holistic patient care. Centers of excellence offering highly specialized treatment to patients with post-acute infection sequlae and/or related disorders following acute respiratory infections may be a significant step forward. These centers could play a key role in optimizing diagnostic and treatment processes during the initial phase of respiratory infections, implementing effective follow-up procedures, systematically enrolling patients in research studies, and analyzing outcomes. This approach would establish a foundation for continuous improvement in patient care and support the design of future health care structures tailored to patients´ needs.

### Limitations

A limitation of this article is the lack of a consistent definition of post-acute infection sequelae, which reduces the comparability of different studies. ARIs and their complications were not diagnosed in a standardized way. This may have biased frequency of both post-acute infection sequelae and comorbidities. Due to multiple terms for post-acute infection sequelae, studies addressing this topic might have been missed. The diagnosis of pulmonary fibrosis is typically based on imaging interpretations without histological confirmation. However, insights gained from the COVID-19 pandemic have shown that lung injuries initially classified as fibrosis following acute infection can improve over time, suggesting caution in using the term “lung fibrosis” without long-term follow-up. The absence of pre-infection pulmonary function values or imaging reports in many studies necessitates cautious interpretation of specific consequences on lung function or imaging findings. Specific post-infectious sequelae following ARDS and mechanical ventilation cannot be distinguished from a Post-Intensive-Care-Syndrome. Finally, many studies included a small number of patients without uninfected controls and were retrospective in nature.

## Conclusion

Non-COVID-19 ARIs are associated with post-acute infection sequelae and the development of multiple complications, including cardiovascular, neurological, secondary pulmonary, psychiatric, and cognitive disorders, and chronic fatigue. These long-term sequelae of respiratory tract infections are of critical importance for patients as they significantly impact both the life span and quality of life of affected individuals, while also placing a burden on society.

## Data Availability

No datasets were generated or analysed during the current study.

## Abbreviations

ARDS: Acute respiratory distress syndrome
ARI: Acute respiratory infection
CAP: Community-acquired pneumonia
CMV: Cytomegalievirus
DLCO: Diffusing capacity of the lungs for carbon monoxide
EQ5D: EuroQol-5 Dimension
FEV1: Forced expiratory volume in the first second
FVC: Forced vital capacity
HIV: Human immunodeficiency virus
HR: Hazard Ratio
HRQoL: Health-related quality of life
MERS-CoV: Middle East respiratory syndrome coronavirus
SF-12: Medical Outcomes Study 12-Item Short Form
SF-36: Medical Outcomes Study Short Form 36-item health survey
Post-acute infection sequelae: Sequelae after acute respiratory infections excluding long COVID
PTSD: Posttraumatic stress disorder
RSV: Respiratory syncytial virus
RTI: Respiratory tract infections
SARS: Severe acute respiratory syndrome
SARS-CoV-1: Severe acute respiratory syndrome coronavirus 1
SARS-CoV-2: Severe acute respiratory syndrome coronavirus 2
SF-12: Medical Outcomes Study 12-Item Short Form
SF-36: Medical Outcomes Study Short Form 36-item health survey
TLC: Total lung capacity
